# The Role of Glucose–Lymphocyte Ratio in Evaluating the Severity of Coronary Artery Disease

**DOI:** 10.3390/jcm13226711

**Published:** 2024-11-08

**Authors:** Faruk Serhatlioglu, Zeki Cetinkaya, Yucel Yilmaz

**Affiliations:** 1 Department of Cardiovascular Surgery, Faculty of Medicine, Nigde Omer Halisdemir University, Nigde 51100, Turkey; faruks@erciyes.edu.tr; 2 Department of Cardiology, Ministry of Health, Elazıg Fethi Sekin City Hospital, Elazig 23280, Turkey; zeki2387@gmail.com; 3 Department of Cardiology, Kayseri City Training and Research Hospital, University of Health Sciences, Kayseri 38080, Turkey

**Keywords:** inflammation, glucose/lymphocyte ratio, chronic coronary syndrome, coronary artery disease severity

## Abstract

**Background:** Recently, a new inflammatory and prognostic marker called glucose/lymphocyte ratio (GLR) has been used in patients with coronary artery disease. In this study, we analyzed the correlation between GLR and coronary artery disease (CAD) severity in patients with chronic coronary syndrome (CCS). **Methods:** The study included 341 patients with CCS who underwent coronary angiography and documented coronary stenosis of 50% or more in at least one major coronary artery and 437 individuals with coronary atherosclerosis between 1% and 50% or no coronary atherosclerosis (control group). Blood samples for GLR and other laboratory parameters were obtained from all patients on admission. GLR was obtained by dividing the glucose level by the lymphocyte count. **Results:** There were more patients with diabetes mellitus (DM) in the critical CAD group, and glucose levels (*p* < 0.001), neutrophil counts (*p* < 0.001), C-reactive protein (CRP) levels (*p* < 0.001), neutrophil/lymphocyte ratio (NLR) (*p* < 0.001), platelet/lymphocyte ratio (PLR) (*p* < 0.001), and GLR (*p* < 0.001) were higher. In contrast, lymphocyte counts were lower (*p* < 0.034). Multivariate logistic regression analysis showed that DM and high CRP were independent predictors of critical CAD (*p* = 0.004 and *p* = 0.048, respectively). However, high GLR was found to be an independent predictor of critical CAD (*p* < 0.001). **Conclusions:** GLR, a simple and easily measured marker, has shown strong predictive value for CAD severity in CCS patients.

## 1. Introduction

Chronic coronary syndrome (CCS) is a heterogeneous group of diseases that includes obstructive and nonobstructive coronary artery disease (CAD), with or without previous myocardial infarction (MI) or revascularization. Although deaths from cardiovascular disease (CVD) have decreased in recent decades, partly due to better care of patients with acute MI, it remains the leading cause of death worldwide [1]. Also, the fact that CVD is one of the main causes of increasing health costs has led to a special focus on this disease group [2,3]. CCS is the first symptom in approximately 50% of all CAD cases. It is usually caused by occlusion of at least one large epicardial coronary artery or one of its branches by atheromatous plaque [4].

Atherosclerosis involves many highly interrelated processes, including platelet activation, thrombosis, lipid disorders, endothelial dysfunction, oxidative stress, inflammation, altered matrix metabolism, vascular smooth cell activation, remodeling, and genetic factors [5]. Atherosclerosis is also defined as a low-grade, non-infectious, systemic chronic inflammatory condition associated with age, psychology, environment, lifestyle, and resolution of acute inflammation [6]. In other words, chronic inflammation is involved in the whole process of the formation and development of atherosclerosis and is the core of atherosclerosis.

The activation of inflammatory pathways and the involvement of immune system cells such as neutrophils, lymphocytes, monocytes, and macrophages contribute to the complexity of inflammation. Therefore, different studies have shown that the presence and degree of chronic inflammation are reflected by the number and percentage of cells associated with the immune system [7,8]. Markers such as red blood cell distribution width (RDW), mean platelet volume (MPV), neutrophil/lymphocyte ratio (NLR), platelet/lymphocyte ratio (PLR), and monocyte/lymphocyte ratio (MLR) are accessible and cost-effective markers of inflammation [8,9,10,11]. CAD is known to be characterized by a high inflammatory burden, and several studies have shown that these inflammatory parameters are associated with the severity and prognosis of CAD [11,12,13,14].

Recently, a new inflammation parameter, glucose/lymphocyte ratio (GLR), has been introduced. Although it has proven to be effective in predicting prognosis in various cancer types, there are limited studies to evaluate its efficacy in CAD and other diseases [15,16,17,18,19,20,21,22,23,24]. While studies investigating the relationship between GLR and CAD are already very limited in the literature, no data have shown the relationship between GLR and CAD severity. This study aimed to investigate the association between GLR and the prevalence and severity of CAD as defined by the Synergy Between Percutaneous Coronary Intervention with TAXUS and Cardiac Surgery [SYNTAX score (SxS)] in patients with CCS.

## 2. Materials and Methods

This retrospective study was conducted from January 2018 to July 2022, and it included 778 patients who underwent coronary angiography (CAG). The study protocol was approved by our local ethics committee.

According to CAG, whether the stenosis of one coronary artery exceeds 50%, 341 patients with CAD were selected as the CAD group, and 437 patients, including coronary atherosclerosis with 1–50% stenosis and no coronary atherosclerosis as the non-CAD group.

Patients with any of the following criteria were excluded from the study: patients with a history of CAD with chronic low-grade inflammation, including those with/without coronary revascularization (patients with a history of medical therapy, coronary artery bypass grafting (CABG), or percutaneous coronary intervention (PCI)); those with highly inflammatory conditions such as acute coronary syndrome (ACS) and history of acute stroke, and patient with heart failure; patients with organ failure accompanied by high levels of inflammation, including kidney, liver, and lung failure; patients with hematological diseases affecting blood cells; and patients with malignancy, as well as those with a history of active infection/systemic inflammatory disease/autoimmune disease affecting the immune system.

Patient features, comorbidities [such as hypertension (HT), diabetes mellitus (DM)], and laboratory data were all variables in this research. The presence of classical cardiovascular risk factors, including age, gender, DM, HT, dyslipidemia, and smoking, was assessed.

HT diagnosis was defined according to the 2018 ESC guidelines. Diagnosis was made by taking two or more measurements obtained in two or more visits with appropriate equipment (cut-off values were determined as systolic blood pressure (SBP) 140 mmHg and/or diastolic blood pressure (DBP) 90 mmHg in-office measurements, regardless of age, gender, and comorbidities). In addition, when ambulatory blood pressure measurement was used for diagnosis, cut-off values were accepted as slightly lower (135/85 mmHg). However, being on medication as defined in the guidelines was also described as HT [25].

DM is defined as fasting blood glucose ≥126 mg/dL after at least 8 h of fasting or random plasma glucose ≥200 mg/dL. However, those previously treated with a history of DM were included in this diagnosis (with diet, oral medications, or insulin) [26].

Dyslipidemia was defined as those with low-density lipoprotein cholesterol levels > 130 mg/dL. In addition, patients previously diagnosed with hypercholesterolemia and treated were evaluated under this diagnosis [27].

Smokers were defined as those who smoked regularly (≥1 year).

### 2.1. Transthoracic Echocardiography

All participants included in the study (patient/control group) underwent transthoracic echocardiography. All measurements were made with the Vivid 7 device (with a 3.5 MHz transducer, GE Medical System, Horten, Norway). Valve structure/function and ventricular ejection fraction (LVEF) were evaluated with two-dimensional echocardiographic measurements. These evaluations were also confirmed with the apical four-chamber view (Simpson method and color Doppler echocardiography). Views with better image quality and/or better evaluations were accepted as correct. When necessary, the results were confirmed by a second cardiologist.

### 2.2. Laboratory Analysis

Antecubital venous blood samples were collected from all participants for laboratory analysis between 8 and 10 a.m. after a 12 h fast before CAG. Blood samples collected for the evaluation of hematological parameters were placed in tripotassium EDTA-based anticoagulant tubes. If hematological samples could not be analyzed within the first 30 min after sampling, they were stored at 4 °C, and then the complete and component counts of hemoglobin, platelets and neutrophils, monocytes and lymphocytes were analyzed with the Sysmex K-1000 autoanalyzer (Sysmex K-1000 Hematology Analyzer, Guangzhou, China). All venous blood samples collected for all routine biochemical tests (including glucose, BUN, creatine, high sensitivity C-reactive protein (hsCRP), plasma lipid profile, etc.) were collected in tubes containing silicone gel, which facilitates the separation of serum or plasma from cells, and sent to the laboratory for centrifugation as soon as possible to obtain serum or plasma. An autoanalyzer (Roche Diagnostic Modular Systems, Tokyo, Japan) was used to evaluate these samples. The following equation was used to calculate GLR: GLR = Glucose/Lymphocyte. The following equation was used to calculate NLR: NLR = Neutrophil/Lymphocyte. The following equation was used to calculate PLR: PLR = Platelet/Lymphocyte.

### 2.3. Coronary Angiography

All participants underwent CAG within 24 h of hospitalization. Experienced interventional cardiologists blinded to the patient groups and clinical/laboratory characteristics performed the CAG procedure/evaluation. The procedure was performed via the radial or femoral artery approach according to the operator’s request, and coronary arteries/branches were evaluated. Routine Judkins catheters were used for cannulation of the coronary arteries. Left anterior descending (LAD) and circumflex (CX) coronary arteries were evaluated in left caudal, left cranial, right caudal, and right cranial views. Additional views were left to the operator’s request. The right coronary artery (RCA) was evaluated in the left anterior oblique and left cranial views. Obstructive CAD was identified as stenosis exceeding 50% of the diameter of the left main coronary artery (LMCA), LAD, CX, and RCA or branch vessels more than 2.0 mm in diameter.

PCI was performed when necessary after CAG imaging. PCI procedures were performed in the same session and using the same approach route as CAG, and a 7 French guide catheter was used for femoral route preferences and a 6 French guide catheter (Launcher; Medtronic) for radial preferences. Standard techniques were used in PCI treatment. All patients included in the study were treated with stent placement in the first session only in the culprit lesion vessel if they had more than one coronary artery stenosis. Drug-eluting stents were preferred as the stent type. Non-ionic and low-osmolar contrast material (io-hexol, Omnipaque 350 mg/mL; GE Healthcare) was used in all procedures.

All patients received oral aspirin (300 mg) along with P2Y12 antagonists such as clopidogrel (300–600 mg), ticagrerol (180 mg), or prasugrel (60 mg) according to PCI guidelines. Intravenous unfractionated heparin was routinely used in all patients as anticoagulant therapy during PCI procedures. Tirofiban was used according to the operator’s preference.

Based on the result of CAG, the severity of CAD was scored using The SxS, which was calculated as per the latest online version of the software (version 2.1) available on the website (https://syntaxscore.org, accessed date; 23 August 2022). CAG image evaluations and individual SxS of all patients were carried out by two experienced interventional cardiologists who were blinded for the study protocol.

### 2.4. Statistical Analysis

Statistical analyses were performed using SPSS version 21.0 (SPSS Inc., Chicago, IL, USA) software for Windows. The distribution of quantitative variables was checked with the Shapiro–Wilk test. Descriptive data were expressed as mean ± standard deviation and median (interquartile range, IQR), depending on the normality of distribution. The median and IQR were used when the variable was not normally distributed. The independent sample t-test was used for the comparison of normally distributed quantitative variables, and the Mann–Whitney U test was used for the comparison of non-normally distributed quantitative variables. Categorical variables were compared to the χ^2^ test. Correlation analyses were performed using Spearman’s coefficient of correlation. The effects of different variables on the development of CAD were calculated with univariate analysis. For multivariate regression analysis, parameters with a *p* < 0.10 in univariate analysis were included in the model. The predictive values of the GLR, the glucose, and the CRP were estimated by the areas under the receiver operating characteristic curve (ROC). A two-sided *p* < 0.05 was considered significant.

G-power was utilized to determine the sample size. For our investigation, 286 participants at minimum were needed, with an alpha error of. 0.05 and a statistical power of 0.95.

## 3. Results

In the present study, a total of 778 participants who underwent CAG were divided into two groups, critical and non-critical CAD, and the baseline characteristics of the patients are summarized in Table 1. There was no statistical difference between the two groups in terms of age, gender, body mass index (BMI), HT, hyperlipidemia, smoking, and LVEF (*p* < 0.05). However, DM patients were more in the critical CAD group (29.9% vs. 20.1%, *p* = 0.002).

When the groups were compared in terms of laboratory findings, the following values were higher in the critical CAD group compared with the non-critical CAD group; glucose levels (*p* < 0.001), neutrophil counts (*p* < 0.001), CRP levels (*p* < 0.001), NLR (*p* < 0.001), PLR (*p* < 0.001), and GLR (*p* < 0.001). In contrast, the lymphocyte counts were lower in the critical CAD group compared with the non-critical CAD group (*p* < 0.034). Other laboratory findings were similar between the two groups (*p* < 0.05) (Table 2).

Multivariate logistic regression analysis is shown in Table 3. It was performed with variables such as DM, glucose, NLR, PLR, CRP, and GLR, which were shown to be associated with the severity of CAD in univariate analysis. Multivariate logistic regression analysis showed that high GLR values were an independent predictor of critical CAD (OR (Odds Ratio) 1.031, 95% confidence interval (CI): 1.023–1.040, *p* < 0.001). However, DM and high CRP were also shown to be additional independent predictors of critical CAD (OR 1.700, 95% CI 1.0189–2.431, *p* = 0.004 and OR 1.035, 95% CI 1.000–1.072, *p* = 0.048, respectively).

**Figure 1:**
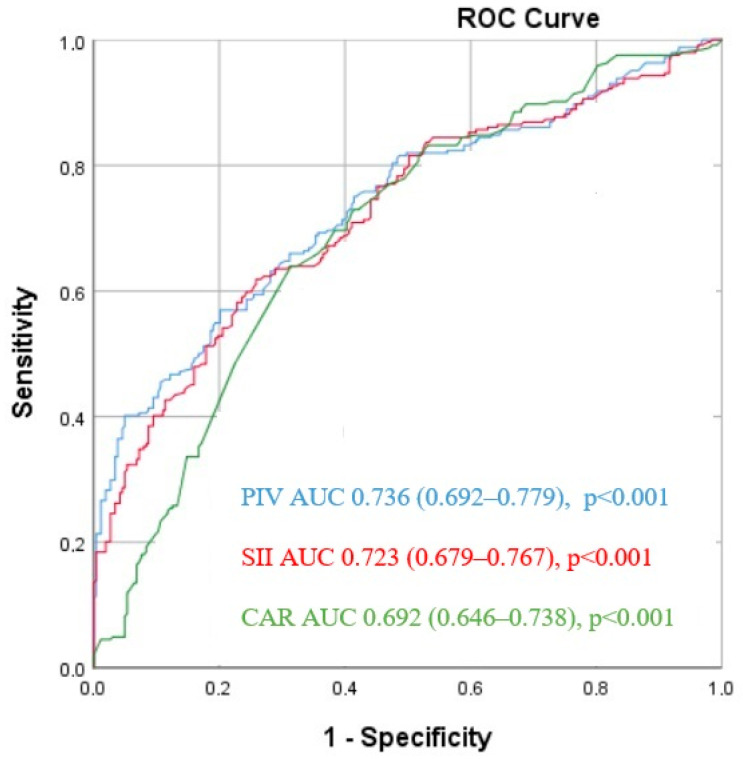
Receiver operating characteristic (ROC) curves for glucose/lymphocyte ratio (GLR), glucose, and high sensitive C reactive protein (CRP) for predicting coronary artery disease severity.

The SxS was 19 + 8 in the critical CAD group. In correlation analysis, while SxS was well associated with GLR (r = 0.744, *p* < 0.001)) and glucose (r = 0.470, *p* <0.001), CRP was found to be non-significant (*p* > 0.05) (Figure 1).

ROC analysis demonstrated that the best cut-off value of 41.2 for GLR was to predict the critical CAD with 83.9% sensitivity and 73% specificity (area under ROC curve = 0.801 (95% CI: 0.765–0.837), *p* < 0.001). For glucose, the best cut-off value of 93.5 predicted the critical CAD with a sensitivity of 65.4% and specificity of 59.1%, and the area under the curve (AUC) was 0.688 (95% CI: 0.650–0.726; *p* < 0.001). For CRP, the best cut-off value of 2.99 predicted the critical CAD with a sensitivity of 70.7% and specificity of 52.9%, and the area under the curve (AUC) was 0.620 (95% CI: 0.581–0.629; *p* < 0.001) (Figure 1). ROC analysis demonstrated that the best cut-off value of 41.2 for GLR was to predict the critical CAD with 83.9% sensitivity and 73% specificity. That is, individuals with a GLR value above 41.2 are considered to have a higher probability of having critical CAD. This means that 83.9% of individuals with critical CAD were correctly identified with a GLR value above 41.2. That is, the rate of identifying individuals with CAD is high. This rate also indicates that 73% of individuals without critical CAD were correctly classified as having a GLR value below 41.2. In other words, the false positive rate (individuals without disease incorrectly classified as having disease) is low.

## 4. Discussion

Our study revealed a higher GLR in patients with severe CAD who underwent CAG for CCS compared with patients with non-critical stenosis and/or no stenosis. Furthermore, GLR was found to be associated with increased coronary atherosclerotic burden as calculated by SxS. Moreover, a significant correlation between GLR levels and SxS was reported.

People with angina pectoris have a higher risk of other CVDs and mortality compared with people without angina [28]. CAD is defined as a pathological condition in which atherosclerosis, usually observed as plaque formation, affects epicardial arteries [29]. As atherosclerosis worsens, the progression of the plaque mass into the lumen can lead to hemodynamic obstruction and angina. Inflammation contributes significantly to the development of atherosclerosis, and the role of inflammation in the etiology of atherosclerosis was confirmed by Chen et al. in a genome-wide meta-analysis of 11.6 million variants [30]. CAD risk factors also damage the endothelium and cause endothelial dysfunction. In the pathogenesis of different stages of CAD, metabolic pathways of lipids, amino acids and carbohydrates from atheroma initiation to progression are associated with the resulting inflammation, endothelial dysfunction, shear stress, and oxidative stress [31]. Elevated markers of inflammation are linked to worse clinical outcomes in CAD, as chronic inflammation plays a role in all stages of atherosclerosis [32].

Attempts to classify the risk of patients with coronary lesions began with the first descriptions of such lesions. Many classification methods have been used over the last 50 years, including the Gensini score. Although these classifications are predominantly based on the number, location, and percentage of coronary obstruction by atherosclerotic plaques, they are sometimes not clinically useful. SxS is also based on the anatomical complexity of plaques in the coronary arteries and is a tool for assessing major adverse cardiovascular events (MACEs) by stratifying into low, intermediate and high-risk groups [33]. Higher SxS values represent a greater therapeutic challenge and a worse patient prognosis [33,34]. Increased SxS is associated with increased coronary atherosclerotic burden, and this has been supported by studies [35,36].

Inflammation is classically defined as the body’s response to cope with injuries [37]. Although this adaptive response is important for tissue repair in the short term, it is thought to cause more harm than good in the long term in many diseases, including metabolic diseases [38]. Although many of the same inflammatory mediators are found in diseases such as obesity and DM, few or sometimes none of the classic features of inflammation (tumor, rubor, dolor, color) have been observed. Therefore, a different injury response or subclass of inflammation has been described as ‘meta-inflammation’ (metabolically triggered inflammation) (also called ‘low-grade’ or ‘chronic’) [38,39]. Whereas the strong and short-lived conventional immune response ends soon after the pathogens are eliminated, the presence of meta-inflammation can last much longer and can severely damage the physiological functions of the tissues involved and trigger various diseases. An unintended consequence of hyperglycemia is associated with this ‘meta-inflammation’ [40].

Studies with GLR are very limited in the literature. Zhong et al. [24] and Yang et al. [20] found a correlation between GLR and mortality in pancreatic cancer patients and colorectal cancer patients. Ni et al. [19] suggested that GLR could be used to predict survival after laparoscopic surgery in renal cell carcinoma. Chen et al. [15] claimed that higher serum GLR levels may be an independent prognostic factor for all-cause and CVD-related mortality in patients undergoing peritoneal dialysis. Zhang et al. [23] showed that GLR can be used as an indicator of in-hospital mortality in patients followed up in the intensive care unit with a diagnosis of acute respiratory dystheresis. Yang et al. [41] reported that as the GLR increased, the in-hospital mortality rate and intensive care mortality rate of patients with non-traumatic cerebral hemorrhage gradually increased in a linear relationship. In other words, a high GLR may also be associated with adverse outcomes such as prolonged hospitalization or increased mortality risk in patients requiring intensive care.

A review of the literature revealed only one study on GLR in patients with CVD. Liu et al. [18] reported 13.74% mortality in a coronary intensive care unit study of approximately 3400 patients with acute MI. In their results, they showed that GLR levels based on glucose and lymphocyte count alone may be a potential predictor for the prognosis of acute MI and can be used for early risk stratification of clinically high-risk populations. That is, they showed that higher GLR may be associated with worse cardiovascular outcomes, i.e., more severe CAD. This ratio may help assess the patient’s prognosis, especially in cases such as ACS or MI.

In our study, we found that GLR levels were higher in patients with more severe CAD, suggesting that GLR is associated with higher levels of inflammation. Chronic inflammation is known to lead to the formation of atherosclerotic plaque and the destabilization and rupture of plaques. This can lead to CAD or increase the severity of existing disease. Elevated GLR may also indicate increased inflammation and stress in the body. Kaya et al. [42] showed that there was a relationship between CAD severity and NLR in patients with CCS. Karabağ et al. [43] similarly found a relationship between CAR and CAD severity in their study. Candemir et al. [44] also found a correlation between SII and SxS in patients with CCS. Although there are not enough studies in the literature to support this issue, considering the studies conducted with other inflammation markers such as NLR, MLR, CAR, and SII, it can be considered that we supported this issue with another inflammation marker such as GLR in our study [42,43,44,45,46]. However, there are some studies showing that GLR is better than inflammation parameters such as NLR and PLR, which are almost routinely used. Navarro et al. [47], in their study evaluating postoperative disease-free survival and death in patients with gallbladder cancer, showed that GLR is a better indicator than PLR/MLR and NLR. Zhong et al. [24] and Chen et al. [16], in their studies (in the patients with pancreatic cancer and acute pancreatitis, respectively), similarly concluded that all three parameters are not as effective as GLR in evaluating prognosis. In our study, when GLR, NLR, and PLR were evaluated, only GLR was shown to be an independent variable, which is consistent with the literature.

The association of DM and CRP with atherosclerosis and CAD is an undeniable fact, and studies are showing that these 2 variables are associated with CAD prevalence [45,46,48,49,50,51]. CRP is a marker of inflammation frequently used in clinical practice. Serum CRP, an acute-phase protein from the liver, is elevated in response to inflammation and has been extensively studied in patients with CAD. It may assess increased cardiovascular risk for individuals with a history of atherosclerosis [43,52]. Furthermore, high CRP levels may predict the likelihood of future MI and stroke [53]. Statins have the potential to reduce CRP levels independently of their cholesterol-lowering effects, which is hypothesized to make CRP a valuable test to re-evaluate individuals at risk for future cardiovascular events [53,54]. Furthermore, unstable angina patients with high CRP levels at discharge are associated with a higher risk of recurrent cardiovascular events or MI within one year [55]. Similarly, high CRP levels at hospital admission for NSTEMI have been reported to be associated with a higher probability of death over a follow-up period of approximately 2 years [56]. Additionally, high CRP levels have been shown to be associated with the severity of coronary involvement in patients with stable CAD [50]. DM is associated with an increased risk of cardiovascular complications after the first year of acute cardiovascular events. In addition to increased long-term mortality, there is also an increased risk of hospitalization for heart failure and reinfarction [57]. Newly discovered glucose intolerance in patients diagnosed with CAD has been found to predict cardiovascular mortality and morbidity. Furthermore, elevated plasma glucose or HbA1c in ACS is associated with a worse prognosis [58,59]. Coronary artery disease is often more prevalent and widespread when DM is present, and the tendency for restenosis, stent thrombosis, and graft occlusion is increased, especially in those treated with insulin [58,60,61,62]. Insulin resistance is important in the pathogenesis of atherosclerosis [63,64,65]. Insulin resistance has been hypothesized to be associated with low-grade inflammation, and insulin resistance may lead to endothelial dysfunction [66,67,68]. In addition, insulin resistance is associated with increased activity of the sympathetic nervous system and impaired cardiac autonomic function [69,70]. Some studies have shown that poor glycemic control and/or postprandial hyperglycemia predict the risk of CAD [71,72,73,74,75]. Hyperglycemia is involved in the pathogenesis of CAD independently of hyperinsulinemia. Hyperglycemia appears to be involved in all stages of the atherosclerotic process. It reduces endothelium-dependent vasodilation [76]. It can glycate proteins and cause microvascular damage [77,78]. It may also potentiate the development of atherosclerosis by exerting direct toxic effects on the vascular system. It is also associated with endothelial dysfunction or apoptosis, hypercoagulability, increased expression of adhesion molecules (VCAMs/ICAMs), increased binding of monocytes to the endothelium, and increased inflammatory response [79,80]. In our study, we found that CCS patients with DM had a higher prevalence of CAD in accordance with the literature. Also, in accordance with the literature, patients with higher CRP levels had higher SxS in our study. This naturally re-supported the association between CRP, a key inflammatory marker, and CAD prevalence.

There is clear experimental evidence that inflammatory mediators can trigger insulin resistance in cells ‘by themselves’. While this may be true for selected conditions with inflammation, such as severe infections, burns, and trauma, there is no evidence to suggest that the same is not true for other chronic metabolic disorders [38]. Nutrient excesses such as glucose and lipids not only induce metabolic disorders but also activate inflammation cascades using similar pathways. This, in turn, causes further metabolic disturbances, resulting in a vicious cycle. One or two mediators mediating these mechanisms may be the following. Nutrient excess triggers inflammatory mediators at the cellular level and stress in the intracellular endoplasmic reticulum (ER) [38]. ER stress itself may cause an increase in Tumour Necrosis Factor-α expression or perhaps more generalized inflammatory responses [81]. A similar situation can occur for free oxygen radicals, which can arise from mitochondria and/or the ER. This causes more ER stress, inhibits insulin action, and generates more reactive oxygen species, which in turn produces broader inflammatory responses [82,83]. In a study of patients presenting with CCS, 36% of patients had impaired glucose tolerance, and 22% had newly diagnosed diabetes. These rates will be higher in acute events [84]. Furthermore, hyperglycemia promotes a prethrombotic state, increases inflammation and sympathetic nervous system activity, worsens endothelial function, causes oxidative stress for the release of reactive oxygen species, and thus exacerbates coronary artery damage [85,86].

Lymphocytes, which are involved in the regulatory pathway of the immune system, are inversely associated with inflammation and play a crucial role in the process of atherosclerosis by regulating the inflammatory response [87,88]. Lymphocytes have an active role in the anti-inflammatory response by enhancing the immune response and regulating serum levels of catecholamines and cortisol during the systemic stress response [89,90]. Inflammation contributes to atherosclerotic plaque formation and progression; it is regulated by immune cells, cytokines and other biomedical markers and may increase atherosclerotic plaque progression and CAD development [91,92]. Lymphopenia may occur due to physiological stereotypes and decreased cell production and/or increased cell apoptosis or redistribution of cells at the tissue level. Increased apoptosis of lymphocytes in an atherosclerotic plaque may result in the progression of plaque development and/or plaque destabilization [93,94]. On the other hand, cumulative evidence has shown that lymphocytes may indicate the nutritional status of patients [95].

The exact mechanism for the close association between increased GLR and CAD severity is largely unknown. It is not surprising that this new parameter obtained by dividing glucose and lymphocyte levels is a more sensitive indicator than these two variables alone. This is because the combination of these parameters into a single index (combining high glucose with low lymphocyte count) is a more sensitive parameter for predicting inflammation than glucose and lymphocytes alone due to the opposing aspects of each marker. Many other inflammation parameters are obtained either by dividing hematologic parameters among themselves (NLR, PLR, MLR, SII etc.) or by dividing metabolic parameters (CAR, TGR etc.) among themselves. This parameter was obtained by the ratio of one metabolic parameter to one hematologic parameter. Since GLR represents the activity of inflammatory and thrombotic processes, it can be speculated that it is more significant than other combined parameters. In our study, it seems to be a better predictor than NLR and PLR.

This study has some limitations. Our study was conducted with a relatively small number of patients. It is also a single-center and retrospective study. This may limit the generalization of the study to the entire population. GLR levels were calculated only at the time of hospitalization. Since the patients were not followed up, information on their changes over time and their effects is limited. Another limitation is that parameters affecting atherosclerosis, such as TGF, NO, and VEGF, were not evaluated in this study. Lastly, there was no long-term follow-up in our study. In addition, the results found in this study do not imply causality due to the nature of the correlation test.

In conclusion, it can be said that there is a potential relationship between GLR and the severity of coronary artery disease, and this ratio can be used in the evaluation of CAD and prediction of its prognosis. However, it should be kept in mind that the number of studies on this subject is limited, and more research is needed.

## Figures and Tables

**Table 1:** Demographic characteristics of the study populations.

	Coronary Artery Disease		
	Non-Critical	Critical	*p* Value
Variables	(*n* = 437)	(*n* = 341)	-
Age (years) ^a^	57 (41–67)	57 (48–64)	0.155
Men gender (n, %)	234 (53.4%)	190 (55.7%)	0.546
BMI (kg/m^2^)	28.45 ± 2.01	29.70 ± 1.6	0.871
Diabetes Mellitus (*n*, %)	88 (20.1%)	102 (29.9%)	0.002
Hypertension (*n*, %)	182 (41.6%)	152 (44.5%)	0.413
Hypercholesterolemia (*n*, %)	89 (20.3%)	80 (23.4%)	0.299
Current smoker (*n*, %)	76 (17%)	68 (19%)	0.363
LVEF (%) ^b^	56 (52–63)	55 (53–60)	0.342
SYNTAX score ^b^	-	19 + 8	-

a; median-IQR, b; mean ± standard deviation; LVEF: left ventricular ejection fraction; BMI: body mass index.

**Table 2:** Evaluation of laboratory findings in patient groups with and without critical CAD.

	Coronary Artery Disease		
	Non-Critical	Critical	*p* Value
Number of Patients	(*n* = 437)	(*n* = 341)	
Glucose (mg/dL) ^a^	89 (78–106)	113 (88–160)	<0.001
Creatinine (mg/dL) ^b^	1.1 + 4	0.96 + 0.6	0.540
AST (U/L) ^b^	24.3 + 16	24.6 + 17	0.844
ALT (U/L) ^b^	23.6 + 18	26.2 + 16	0.157
Total cholesterol (mg/dL) ^b^	197.2 + 46.4	199.4 + 40	0.502
High-density lipoprotein cholesterol (mg/dL) ^b^	48.7 + 12	48.5 + 12	0.797
Low-density lipoprotein cholesterol (mg/dL) ^b^	117 + 37	120 + 35	0.221
Triglyceride (mg/dL) ^b^	155.4 + 87	159.1 + 87	0.565
Hemoglobin (mg/dL) ^b^	14.0 + 2	14.3 + 2.2	0.452
Platelets (10^3^/µL) ^a^	242 (190–301)	230 (190–293)	0.189
WBC (10^3^/µL) ^a^	6.9 (4.8–10)	7.4 (5.5–10)	0.112
Neutrophil (10^3^/µL) ^a^	3.9 (2–7.4)	4.2 (3–6.9)	0.010
Lymphocyte (10^3^/µL) ^a^	2.5 (2.1–3)	2 (1.4–2.8)	0.034
NLR ^a^	1.4 (0.7–3)	2.1 (1.2–3.7)	<0.001
PLR ^a^	92 (69–122)	110 (73–172)	<0.001
GLR ^a^	34.7 (28.2–42.4)	63.7 (49.4–78.1)	<0.001
hs-CRP^a^	2.88 (1.1–6.3)	4 (2.5–7.3)	<0.001

a; median-IQR, b; mean ± standard deviation; WBC: white blood cell; NLR: neutrophil-to-lymphocyte ratio; PLR: platelets-to-lymphocyte ratio; GLR: glucose-to-lymphocyte ratio; hs-CRP: high-sensitive C-reactive protein.

**Table 3:** Univariate and multivariate of predictors of critical coronary artery disease.

	Multivariate Analysis			Univariate Analysis		
	Odds Ratio	95% CI	*p* Value	Odds Ratio	95% CI	*p* Value
Diabetes Mellitus	1.693	1.218–2.352	0.002	1.700	1.189–2.431	0.004
Glucose	1.012	1.009–1.016	<0.001			
GLR	1.018	1.012–1.024	<0.001	1.031	1.023–1.040	<0.001
NLR	1.119	1.053–1.188	<0.001			
PLR	1.007	1.005–1.010	<0.001			
hs-CRP	1.037	1.005–1.070	0.023	1.035	1.000–1.072	0.048

CI: confident interval; GLR: glucose-to-lymphocyte ratio; NLR: neutrophil-to-lymphocyte ratio; PLR: platelets-to-lymphocyte ratio; hs-CRP: high-sensitive C-reactive protein.

## Data Availability

All authors are guarantors of the study and therefore have full access to all data in the study and are responsible for the integrity of the data and the accuracy of the data analysis.
